# Bifunctional Surfaces With Immobilized Antibodies and Bioactive Peptides Mediate Selective Capture and Proliferation of Endothelial Colony‐Forming Cells

**DOI:** 10.1002/adhm.202505092

**Published:** 2026-04-09

**Authors:** Hugo A. Level, Marc‐Antoine Campeau, Mohamed A. Elkhodiry, Gaétan Laroche, Jean‐François Tanguay, Corinne A. Hoesli

**Affiliations:** ^1^ Department of Chemical Engineering McGill University Montreal Canada; ^2^ Centre de Recherche du CHU de Québec & Département de Génie des Mines, des, Matériaux et de la Métallurgie Université Laval Québec Canada; ^3^ Montreal Heart Institute, University of Montreal Montreal Canada; ^4^ Department of Biomedical Engineering Research Institute of the McGill University Health Center Montreal Canada

**Keywords:** antibodies, cell capture, cellular therapy, endothelial colony‐forming cells, extracellular matrix, microcarriers, peptides, surface modification

## Abstract

Endothelial colony‐forming cells (ECFCs) are of significant interest in vascular biomaterials engineering and cell‐based therapies for cardiovascular diseases. Efficient isolation and expansion of ECFCs on polymeric surfaces either in vitro or in situ in the case of implants could significantly advance ECFC‐based therapies. We present a novel bifunctional surface modification strategy combining oriented antibodies and extracellular matrix‐derived peptides to selectively capture ECFCs on surfaces and then mediate firm adhesion, spreading and proliferation. Studies were conducted with anti‐CD309 antibodies and custom RGD peptides, previously shown to respectively enable ECFC capture and clonal expansion. To aid in oriented antibody immobilization on surfaces while modulating antibody‐RGD surface concentrations, Fc‐binding peptides and RGD peptides were co‐immobilized on aminated surfaces using click chemistry, followed by affinity‐mediated antibody immobilization. Bifunctional anti‐CD309 + RGD surfaces selectively captured ECFCs from a mixture with peripheral blood mononuclear cells in dynamic conditions. The presence of RGD peptides significantly enhanced cell spreading and proliferation, leading to additive effects on surface coverage under flow. Proof‐of‐concept studies demonstrated successful adaptation to 3D polystyrene microcarriers, showcasing potential scalability for clinical‐grade cell production. The surface modification scheme provides a versatile and clinically translatable platform for advancing ECFC‐based regenerative therapies.

## Graphical abstract

A tunable bifunctional surface is developed by combining Fc‐binding peptides and RGD motifs to present oriented antibodies alongside integrin‐mediated signaling. The platform selectively captures endothelial colony‐forming cells while promoting their adhesion and proliferation. Application to microcarriers demonstrates scalability, highlighting the potential of this strategy for regenerative medicine and advanced biomaterials engineering.

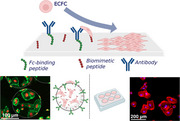

## 1 Introduction

Cell therapies hold significant promise in the treatment of degenerative diseases including cardiovascular conditions, which remain the leading cause of death globally. Different cell populations isolated from peripheral blood have shown promise in promoting neovascularization upon in vitro expansion and transplantation in animal models of heart disease or ischemia [1, 2, 3, 4]. Among these, endothelial colony‐forming cells (ECFCs) residing in the CD31^+^CD34^+^CD146^+^CD309^+^CD45^−^CD14^−^CD133^−^ [5, 6, 7, 8, 9] fraction of peripheral blood mononuclear cells (PBMCs) show great promise in therapeutic revascularization through direct integration in neovasculature [10, 11]. Conventional ECFC culture methods rely on late outgrowth of endothelial colonies on collagen‐coated surfaces directly from peripheral or cord blood samples [12, 13]. However, these methods have several limitations, with a relatively high failure rate (around 30%) and lengthy culture time, with colony formation occurring within weeks after cell seeding [13]. Therefore, selective isolation protocols can be applied prior to plating to maximize yields. Such techniques include microfluidic [14] and magnetic [15] cell separation targeting typical ECFC surface markers such as CD34 or CD31. These cell selection methods introduce additional bioprocessing steps that can lead to cell losses or undesired byproducts (magnetizable particles) which can be problematic in transplantation applications. Animal‐derived protein coatings such as collagen applied after cell selection may contain adventitious agents that are not compatible with current good manufacturing practices (cGMP).

An alternative approach is to directly capture ECFCs on cell culture surfaces for *in vtro* expansion using immobilized antibodies. Antibody immobilization can be achieved by either covalently cross‐linking antibodies to the surface or by taking advantage of bio‐affinity interactions [16]. The latter is especially interesting for controlling the orientation of the antibodies by targeting specific interactions with the fragment crystallizable (Fc) domain on the antibody. Recently, our group demonstrated the feasibility of ECFC capture using oriented anti‐CD309 (VEGF receptor 2) antibodies immobilized via protein G grafted on a polystyrene substrate [17]. We also achieved selective ECFC capture with a smaller Fc‐binding peptide containing the RRGW sequence without the need of larger and more expensive Fc‐binding proteins [18].

This peptide‐mediated antibody interaction gives the opportunity to include other functionalities by grafting two or more peptides before antibody immobilization. As such, instead of animal‐derived protein coatings used for ECFC expansion such as collagen, extracellular matrix (ECM)‐derived peptides can be grafted on surfaces to mediate cell attachment and integrin activation following capture. Linear peptide sequences such as GRGDS are known to promote cell adhesion by interacting with specific integrins, mostly the *α*vβ3 and *α*vβ5 subtypes [19] which are highly expressed in ECFCs [20]. Other sequences such as the collagen‐mimetic GEKGER have demonstrated improved mesenchymal cell adhesion [21]. We previously demonstrated that RGD‐functionalized polystyrene surfaces enable primary ECFC colony formation and increase ECFC clonal expansion as compared to collagen‐coated surfaces [22]. In biomanufacturing applications, using short peptide sequences for surface functionalization offers numerous advantages over larger proteins, including improved thermal stability and an increased scalability for cGMP‐compliant production.

Here, we describe a novel bifunctional surface modification strategy that combines the benefits of selective cell capture with biomimetic signaling to promote selective adhesion, spreading and proliferation of ECFCs. This approach is based on the combination of an Fc‐binding peptide and an ECM‐derived RGD peptide with similar molecular weights in solution that are covalently bound to a polystyrene substrate using click chemistry to create tunable bifunctional surfaces. These engineered surfaces can selectively capture ECFCs from a mixed population of peripheral blood mononuclear cells (PBMCs) and promote subsequent RGD‐mediated cell spreading. More broadly, the method by which antibodies and bioactive peptides are combined on surfaces creates a versatile platform for biomaterials surface engineering.

## 2 Materials and Methods

### 2.1 Surface Modification

**FIGURE 1:**
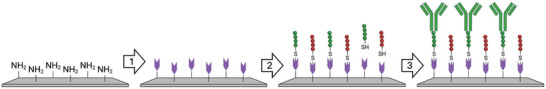
Schematic representation of bifunctional surface modification steps. (Step 1) Surface activation with a heterobifunctional crosslinker (Sulfo‐SMCC) applied to substrates with primary amines. (Step 2) Covalent peptide grafting via maleimide‐thiol "click" chemistry. (Step 3) Affinity‐based antibody immobilization on Fc‐binding RRGW peptides.

The surface reaction scheme is shown in Figure 1, with applied reactants and concentrations described in Table 1. Surface modifications were carried out on aminated polystyrene surfaces from different suppliers (PureCoat Amine, Corning, NY; Biomat, Italy). Unless otherwise mentioned, all reactions and rinsing steps were performed in Dulbecco's phosphate buffer saline (DPBS, Thermo Scientific; 14190) adjusted to pH 7.2. All reactions were performed in static conditions, in the dark and at room temperature with 100 uL/cm^2^. After peptide grafting, surfaces were rinsed, dried and kept for up to 3 days at 4°C prior to experiments. Antibodies were applied immediately before each experiment. For cell studies longer than 2 h, peptide grafted surfaces were sterilized in 70% ethanol for 15 min, and antibody solutions were passed through 0.22 µm syringe filters before immobilization in sterile DPBS.

**TABLE 1:** Surface modification steps and reagent compositions.

Reaction step	Reactant	Incubation time (h)	Concentration/buffer
1	sulfosuccinimidyl 4‐(N‐maleimidomethyl)cyclohexane‐1‐carboxylate (Sulfo‐SMCC; Thermo Scientific; 22322)	1	1 mg/mL in DPBS (pH = 7.2)
2	Single peptides or combinations (all supplied by Biomatik, NJ, USA): CGGGGGRRGW (RRGW) CGKGGRGDS‐NH_2_ (RGD) CG‐K(Biotin)‐GGRGDS‐NH_2_ CG‐K(Trifluoroacetyl)‐GGRGDS‐NH_2_ (RGD‐F) CGKGGRDGS‐NH_2_ (RDG)	2	Peptides were mixed at different ratios DPBS (pH = 7.2). Unless otherwise specified, the total concentration of peptide is 100 µM. For example, 50‐50 RRGW‐RGD represents 50 µM of RRGW and 50 µM of RGD.
3	Mouse IgG_1_ anti‐CD309 antibody (Biolegend; clone A16085H; 393002)	1	5 µg/mL for ELISA 10 µg/mL for all other experiments

### 2.2 XPS Analysis

X‐ray photoelectron spectroscopy was used to confirm peptide grafting on the polystyrene surfaces. Aminated Petri dishes (PureCoat Amine, Corning) were cut into 1cm^2^ coupons that were then functionalized as described above using RGD‐F to enable fluorine atoms detection. Nexsa G2 X‐Ray Photoelectron Spectrometer (Thermo Scientific) was used to collect survey spectra with 200 eV pass energy, 50 ms dwell time and 3 scans. High resolution spectra of sulfur (S2p) were collected with 20 eV pass energy, 50 ms dwell time and 15 scans. All XPS data was analyzed using the Avantage software (Thermo Scientific). On high resolution spectra, peaks were fitted to identify the specific chemical states of carbon and sulfur.

### 2.3 ELISA

The wells of amine‐treated 96 well plates (Biomat, Italy; MT02F4‐AM1) were modified as described in Table 1, up to Step 2. To detect the RGD peptide by ELISA, 1% of the RGD molar ratio was replaced by RGD‐biotin during peptide grafting. After Step 2, surfaces were blocked with a 2% bovine serum albumin (BSA, Sigma; A7906) solution for 15 min. Horseradish peroxidase‐conjugated streptavidin (Streptavidin‐HRP, Abcam; ab7403) at 0.04 µg/mL was then incubated on the surface for 30 min in 0.5% BSA and 0.05% Tween‐20 (Sigma; P9416) prepared in DPBS (DPBS‐T). After 3 rinses with DPBS‐T, the solution was replaced by 3,3′,5,5′‐tetramethylbenzidine (TMB, Thermo Scientific; 00420156) before stopping the reaction with 100 µL of 2N sulfuric acid (Fisher; 831032) in each well. Absorbance was measured on a Benchmark Plus Microplate Spectrophotometer (Bio‐Rad) at 450 nm. For antibody detection, after Step 2, a 2 h blocking step was performed using a 10 mM L‐cysteine solution (Sigma; 30089) in DPBS to remove potential background caused by unreacted maleimides. After Step 3, the surface was blocked with 2% BSA in DPBS for 15 min before detection using horseradish peroxidase (HRP)‐conjugated goat anti‐mouse IgG (H+L) antibody (Invitrogen, G‐21040) applied at 0.1 µg/mL in 0.5% BSA in DPBS‐T. After 3 rinses with DPBS‐T, detection was performed with TMB substrate until developed (15‐30 min) before stopping the reaction with 100 µL of 2N sulfuric acid solution and reading the absorbance at 450 nm. Negative controls without the anti‐CD309 antibody were performed to confirm that the HRP‐conjugated goat anti‐mouse IgG did not interact with the RRGW sequence (data not shown).

### 2.4 Spot Patterning

Aminated polystyrene 6‐well plates (PureCoat Amine, Corning; 356721) were treated with Sulfo‐SMCC. At Step 2 of the reaction scheme, 3 µL spots of RRGW‐RGD applied at 100‐0 to 0–100 ratios were deposited on the surface. Unreacted maleimide groups were blocked with 10 mM L‐cysteine in DPBS for 2h. After rinsing and air drying, 30 µL spots of 10 µg/mL anti‐CD309 antibody solutions in DPBS were added to completely cover the peptide spots. Surfaces were then rinsed with DPBS‐T and blocked with 2% BSA DPBS for 30 min. Finally, all surfaces were reacted with 0.5% BSA DPBS‐T containing either 5 µg/mL Alexa Fluor 488‐labelled streptavidin (Invitrogen; S11223) or 10 µg/mL Alexa Fluor 555‐labelled goat anti‐mouse IgG antibody (Invitrogen; A‐21422) to detect the grafted peptides (RGD‐biotin) or the immobilized anti‐CD309 antibodies, respectively. After rinsing with DPBS‐T, all spots were imaged using an Olympus IX81 inverted fluorescence microscope at 10X magnification. For each condition, 3 images were taken at the edge of the spots.

### 2.5 ECFC Isolation, Characterization, and Culture

ECFCs were isolated and expanded from human peripheral blood based on our previously described isolation protocol [22]. Briefly, blood samples were received from 4 healthy donors under informed consent following a protocol (Study No. A06‐M33‐15A) approved by the Ethics Institutional Review Board at McGill University. Prior to cell experiments, ECFCs were cultured in complete endothelial cell growth medium‐2 (EGM‐2, Lonza, Switzerland; CC‐3162) supplemented with 10% fetal bovine serum (FBS; Fisher; SH3039603) at 37°C and 5% CO_2_ and harvested between passage 2 and 4. For every cell experiment, at least 3 replicates were performed with ECFCs isolated from different donors, both males and females, whose age ranged from 25 to 60 years old. ECFC phenotype (CD31^+^, CD34^+^, CD105^+^, CD144^+^, CD146^+^, CD309^+^, CD14^−^, CD45^−^) was validated for each donor by flow cytometry (Figure S1 and Table S1).

### 2.6 ECFC Adhesion and Spreading in Static Conditions

To study ECFC adhesion under static conditions, the wells of aminated 24‐well plates (PureCoat Amine, Corning; 354723) were modified with combinations of RGD and RRGW peptides before antibody immobilization as described in section 2.1 (Table 1). ECFCs were resuspended in equilibrated (37°C; 5% CO_2_) serum‐free EGM‐2 where FBS was replaced by 0.4% BSA (EGM2‐BSA). Cells were seeded at 5,000 cells/cm^2^ in each well. After either 30 min or 2 h of incubation, surfaces were washed with sterile DPBS, and cells were fixed with 4% paraformaldehyde (PFA; Fisher; BP531) in DPBS for 20 min. After rinsing, the fixed cells were kept protected from light at 4°C in sterile DPBS for up to a week before staining (section 2.11).

### 2.7 ECFC Proliferation in Static Conditions: EdU Assay

To study the early induction of ECFC proliferation, aminated 24‐well plates were modified as described in section 2.1. ECFCs were resuspended in EGM2‐BSA and seeded at 10 000 cells/cm^2^. After 2 h, culture media were replaced by EGM‐2 supplemented with 0.5% FBS and cells were left to growth overnight. An EdU staining proliferation kit (Abcam; ab219801) was used as per the manufacturer's instructions. Briefly, 0.5 mL of culture medium was replaced by 0.5 mL of fresh EGM‐2 containing 0.5% FBS and 40 µM of 5‐ethynyl‐2’‐deoxyuridine (EdU) in each well. After 2 h, wells were rinsed and fixed for 20 min with 4% PFA solution. Immediately after rinsing, cells were permeabilized with 0.1% Triton‐X 100 (Sigma; X100) and internalized EdU was labelled with an iFluor 488 azide dye. Finally, a 10 ug/mL 4',6‐diamidino‐2‐phenylindole (DAPI; Sigma; D9542) solution in DPBS was added in each well for 20 min to counterstain the nuclei of all adhered cells. Cells were imaged and analyzed as described below.

### 2.8 ECFC Capture on Polystyrene Plates Under Flow

ECFC capture was evaluated in modified aminated 6‐well plates (PureCoat Amine, Corning). Prior to surface modification, 6 8‐mm diameter areas were circumscribed under each well, uniformly spaced at 1 cm distance from the well center. At steps 2 and 3 of the reaction scheme (Table 1), peptides or antibody solutions were applied by pipetting 20 µL onto the indicated areas. Modified wells were washed with sterile DPBS and filled with 0.5 mL of equilibrated EGM2‐BSA. An ECFC suspension at 1 × 10^6^ cells/mL was slowly added to the center of the wells (0.5 mL/well). The well plate was then immediately placed on a digital waving rotator (Thermo Scientific; 88882004) at 90 rpm with no tilt angle for 2 h (37°C, 5% CO_2_). After incubation, wells were rapidly washed with equilibrated EGM2‐BSA 3 times to remove non‐adhered cells before fixation with 4% PFA. The fixed surfaces with cells were kept at 4°C in sterile DPBS for up to a week before staining.

### 2.9 ECFC Capture Selectivity

Similarly to the ECFC capture protocol (Section 2.8), small spots in the wells of an aminated 6‐well plate were modified with peptide mixtures before either anti‐CD309 or anti‐CD45 (clone HI30; BD Pharminogen; 555480) antibodies were immobilized on the surfaces to selectively target ECFCs or PBMCs, respectively. PBMCs were isolated from human peripheral blood using SepMate isolation tubes (STEMCELL Technologies, Canada; 85450) and the associated protocol. A 1:1 ECFC to PBMC ratio with 5 × 10^5^ total cells was used for the capture assay as described in Section 2.8. Controls with only ECFCs or PBMCs were performed to confirm the absence of interaction between cell types.

### 2.10 ECFC Capture on Modified Polystyrene Microcarriers

Aminomethyl polystyrene beads (150‐200 µm in diameter) ((aminomethyl)polystyrene beads (70‐90 mesh; Sigma, 515620) were modified in suspension. For each experimental condition, 7 mg of beads (2 cm^2^ equivalent surface) were placed in 1.5 mL microcentrifuge tubes and a volume of 300 µL was used for each reaction (Table 1). Tubes were placed on a tube revolver rotator (Thermo Scientific, 88881001) at 10 rpm to ensure proper mixing. Between each modification step, beads were allowed to settle to remove the supernatant and washed twice with 1 mL of DPBS. ECFC capture assays on microcarriers were performed in sterile 2mL cryotubes (Sarstedt; 72.380). 1 cm^2^ equivalent of treated microcarriers were added to each cryotube. ECFC suspension in equilibrated EGM2‐BSA was added to the cryotubes (1 × 10^5^ cells in 1.5 mL final volume) before incubating the tubes on the tube rotator at 10 rpm for 1 h (37°C, 5% CO_2_). After incubation, the supernatant was quickly removed and the microcarriers were resuspended in equilibrated EGM2‐BSA before transferring to a 100 µm reversible strainer (STEMCELL Technologies, Canada; 27217). The microcarriers were washed twice with 1 mL of medium to remove weakly adhered cells and were transferred to a clean microcentrifuge tube by inverting the strainer. Microcarriers were then resuspended in complete EGM‐2 + 10% FBS and 0.5 cm^2^ equivalent of microcarriers were seeded in suspension 48‐well plates (Sarstedt; 833923500). A premix solution of water‐soluble tetrazolium 1 (WST‐1; Abcam; ab155902) was added to each well at a final concentration of 10% v/v and incubated overnight (37°C, 5% CO_2_). Relative cell capture was evaluated by performing absorbance measurements at 440 nm. For each experimental condition, a small fraction of microcarriers were fixed immediately after capture with 4% PFA in DPBS using a 0.6 mL microcentrifuge tube in order to perform subsequent cell staining and confocal imaging.

### 2.11 Cell Staining and Imaging

For actin and nuclei staining, fixed samples were permeabilized with 0.1% Triton‐X and stained with a solution of 10 µg/mL 4',6‐diamidino‐2‐phenylindole (DAPI; Sigma; D9542 and phalloidin‐iFluor 488 (1:1000; Abcam; ab176753) in DPBS for 30 min at room temperature. After rinsing and filling the wells with 3mL DPBS, 9 images from each condition were obtained at randomized locations using the 10x objective on an Olympus IX81 fluorescence microscope equipped with an automated stage (Prior Scientific Inc., Rockland, MA).

For cell selectivity experiments, ECFCs were stained using PE‐conjugated anti‐CD31 (PECAM‐1) (BD Bioscience; clone WM59; 555446) in 1% DPBS‐BSA followed by DAPI counterstaining.

The images of cells on microcarriers were obtained following a similar staining protocol with SYTOX deep red nucleic acid stain (Thermofisher; S11380) and phalloidin‐iFluor 488. After staining, microcarriers were resuspended in 50 µL of DPBS and transferred to a glass‐bottom petri dish. Finally, 100 µL of Vectashield Vibrance antifade mounting medium (Vector Laboratories; H‐1700) was added on top of the beads and a round 1cm^2^ coverslip was sealed with nail polish on top. Z‐stacks were obtained by laser confocal microscopy using a Zeiss LSM800 microscope. Final images were generated by maximum intensity projection.

### 2.12 Image Analysis

All images were analyzed using the ImageJ software (US National Institutes of Health). Enumeration of total adhered cells was done by counting the number of cell nuclei in each DAPI image using the thresholding and “analyze particles” tools. Proliferative cells were analyzed by counting the number of EdU positive nuclei (green fluorescent channel).

The total cell area was quantified using the fluorescent phalloidin images showing F‐actin filaments by adjusting the signal threshold, masking the positive regions, and calculating the area occupied by all cells using the “ROI manager” tool. Average individual cell surface area was obtained by dividing the total cell coverage by the number of nuclei in each image.

### 2.13 Statistical Analysis

Statistical analyses were performed using the GraphPad Prism 10 software. ELISA and WST‐1 assays were analyzed using a one‐way ANOVA followed by a parametric multiple comparison test (Tukey's range test). For all cell experiments generating numerous datapoints with high donor variability, a non‐parametric paired test (Friedman's test) was used to account for the pairing within each donor. Dunn's non‐parametric multiple comparison method was then applied post‐hoc to observe significant differences among conditions. For ELISA experiments, the JMP Pro 17 software (SAS Institute Inc.) was used to perform linear models between the absorbance and the surface composition. In Figures 4, 5, 6, colored dots represent individual datapoints from images taken, while colored triangles with contours represent the average of all datapoints for a single ECFC donor. Error bars represent the mean ± standard deviation of 3 to 4 ECFC donors (n = number of triangles on plots).

## 3 Results

### 3.1 Characterization of Bifunctional Surfaces

**FIGURE 2:**
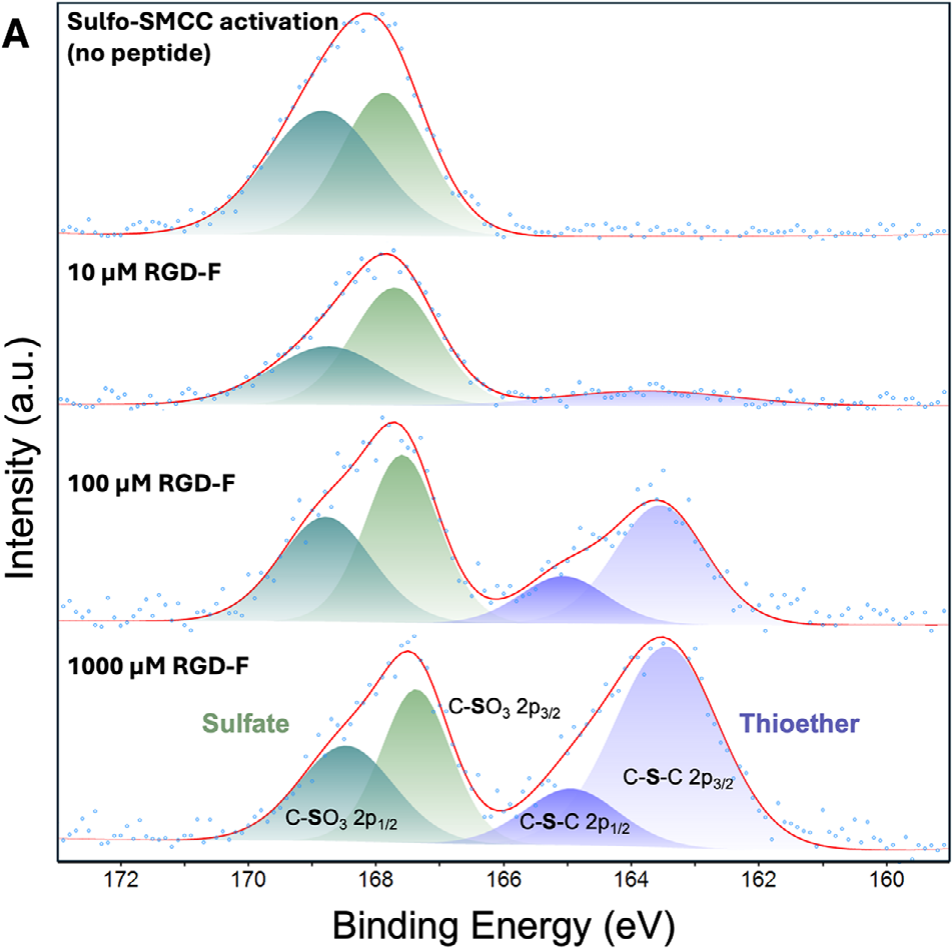
XPS Surface characterization of aminated polystyrene plates functionalized with a fluorinated RGD peptide (RGD‐F). Shown are high resolution spectra of sulfur on modified polystyrene obtained with different RGD‐F concentrations showing the presence of both sulfates (from sulfo‐SMCC) and thiols (from cysteine residues).

The stepwise surface reaction scheme (Figure 1) was monitored by analyzing the surface elemental composition by XPS (Figure 2 and Table 2). Aminated polystyrene surfaces were first treated with Sulfo‐SMCC, which releases SO3^−^ groups that can adsorb on surfaces. As expected, S was detected after this step, in the form of S2p (doublet around 168 eV) in high‐resolution XPS spectra. After covalent grafting of the RGD‐F peptide, a new doublet appeared on the S2p high‐resolution spectra. This doublet has a lower binding energy (around 164 eV), corresponding to the thioether bond formed after maleimide‐thiol coupling. In this experiment, the RGD‐F peptide was chosen instead of RGD as it introduces fluorine on the surface with a trifluoro acetyl group on the lysine residue. As shown in Table 2, an increase in the surface atomic percentage of fluorine as a function of the RGD‐F peptide concentration that reacted with the sulfo‐SMCC was observed. As expected, a positive relationship between the peptide concentration in solution and the fraction of fluorine on surfaces was found which is consistent with proper biomolecule grafting.

**TABLE 2:** Elemental composition (%) after peptide grafting vs controls.

Reaction step	Carbon C 1s	Nitrogen N 1s	Oxygen O 1s	Fluorine F 1s	Sulfur S 2p
Bare aminated polystyrene	74.39	15.03	10.57	0	0
Sulfo‐SMCC	69.25	14.54	15.24	0	0.83
RGD‐F 10µM	69.58	14.80	14.63	0.20	0.53
RGD‐F 100 µM	71.50	13.88	12.99	0.89	0.62
RGD‐F 1000 µM	67.66	15.68	14.58	1.18	0.77

**FIGURE 3:**
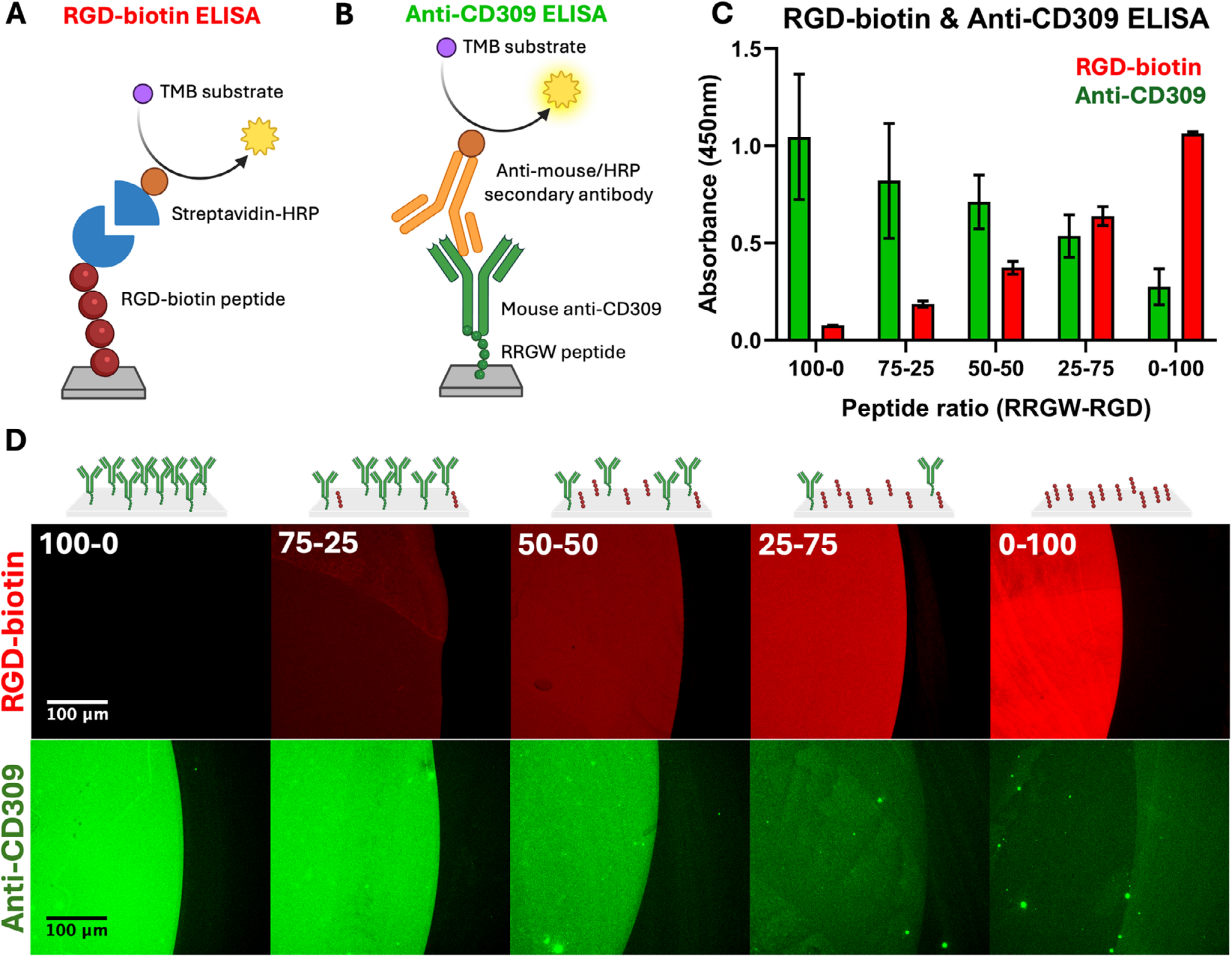
Surface characterization of a highly controllable bifunctional surface obtained by combining biomimetic (RGD) and antibody‐binding (RRGW) peptides in solution. (A) RGD‐biotin ELISA and (B) anti‐CD309 ELISA assay mechanism. (C) Relative absorbance of RGD‐biotin and anti‐CD309 ELISA assays for various peptide ratios showing an inversely proportional relationship between the surface concentration of RGD‐biotin and immobilized antibody on modified surfaces (n=4). (D) Representative images of bifunctional spots on polystyrene for various peptide ratios showing immobilized CD309 antibodies (green) and grafted RGD peptide (red).

Next, combinations of RGD peptides and RRGW peptides were applied to surfaces with the goal of subsequently immobilizing antibodies via Fc interactions with RRGW. The impact of peptide concentrations applied in solutions on the relative grafted peptide density was studied using customized ELISA assays (Figure 3). Different RRGW‐RGD peptide ratios were applied to surfaces (with 1% of the RGD peptides being biotinylated for detection). For 100 µM of total peptide concentration in solution, both ELISA signals correlated linearly with the proportion of respective peptides applied in solution (Figure 3C). A similar linear relationship was found when a recombinant biotinylated VEGFR‐2 protein was reacted with our modified surfaces (Figure S2) to demonstrate the antigen binding capabilities of the bifunctional surfaces. Interestingly, when different total peptide concentrations were applied, ranging from 1 to 1000 µM (Figure S3), the anti‐CD309 ELISA showed saturation at high peptide concentration. This saturation could be due to steric hindrance between antibodies being reached at high RRGW surface concentrations, given the much higher molecular weight and size of antibodies as compared to the peptides used in this study. Thus, further experiments were conducted at 100 µM total peptide concentration in solution. By adapting these ELISA protocols, we also demonstrated that both peptide grafting and antigen recognition remained stable on the surface after 7 days of incubation in EGM‐2 culture medium (Figure S4). The surface characterization results were further confirmed by modifying spots on a polystyrene surface with different peptide ratios, using fluorescently labelled streptavidin and secondary antibodies to detect the RGD‐biotin peptide and immobilized anti‐CD309, respectively. Gradients in the fluorescence signals were concomitant with the trends given by our ELISA protocols (Figure 3D).

### 3.2 Static ECFC‐Surface Interaction Studies

**FIGURE 4:**
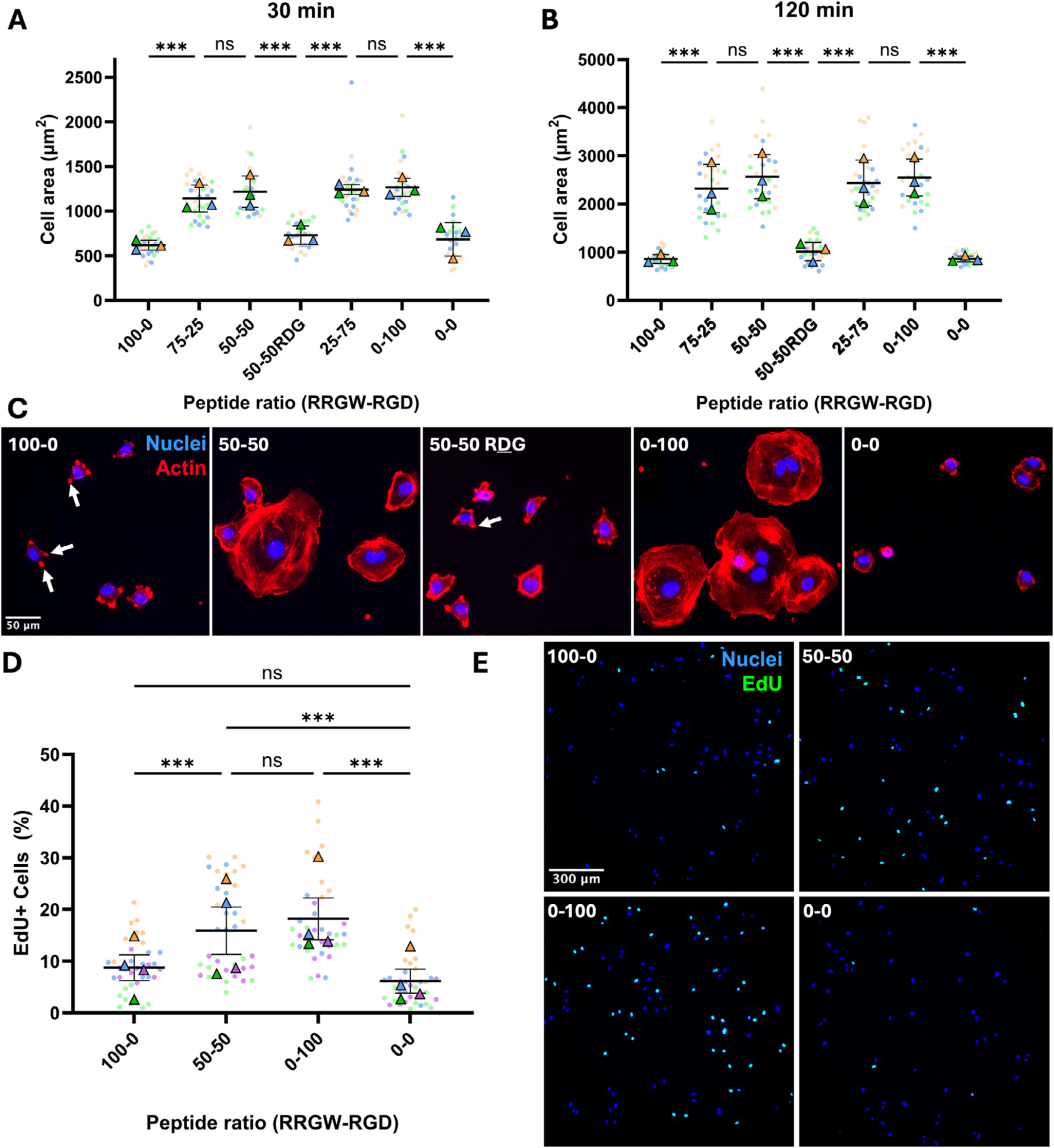
ECFC response to functionalized surfaces in static culture conditions. Average individual cell surface area after 30 min (A) and 120 min (B) incubation demonstrating RGD‐mediated cell spreading (n=3 donors). (C) Representative fluorescence images showing ECFC cytoskeletal organization after 120 min incubation. White arrows indicate lamellipodia‐like extensions induced by the interaction with the antibody in absence of RGD‐mediated integrin signaling. (D) Percentage of proliferative (EdU positive) cells after 24h of incubation on surfaces treated with various peptide ratios (n=4 donors). (E) Representative fluorescence images showing ECFC nuclei after 24h of incubation showing EdU integration.

One critical aspect of the bifunctional surface modification approach relies on the conservation of the biological functionalities of each component. To study the effect of RGD signaling on our bifunctional polystyrene surfaces, ECFCs were seeded at a low density and the average individual cell area was measured after 30 or 120 min in serum‐free static cultures (Figure 4). As early as 30 min, the presence of RGD on the surface significantly increased cell spreading (Figure 4A). This effect became more prominent over time, as ECFCs in contact with RGD‐presenting surfaces continued to spread between 30 and 120 min. This suggests that the presence of immobilized antibodies did not prevent the interactions between ECFCs and the short RGD sequence. Both control surfaces, with either the scrambled RDG peptide or the RRGW and antibodies alone, did not show any significant effect on cell spreading compared to the unmodified polystyrene. There was no significant difference in cell spreading between 25–75 and 0–100 RRGW‐RGD ratios, suggesting that even lower RGD surface concentrations are sufficient to maximize cell spreading in static conditions, despite the presence of RRGW and antibodies. Cytoskeleton staining after 2 h of cell culture presented a well‐developed network of actin filaments within the cells on surfaces functionalized with the ECM‐derived RGD peptide. In contrast, ECFCs on RGD‐free surfaces exhibited a cortical actin distribution, with no visible internal structure (Figure 4C). On surfaces containing immobilized antibodies but lacking RGD peptides, the cells showed protrusions with high actin density (indicated by white arrows). These likely coincide with the localized interactions between immobilized anti‐CD309 antibodies and the cell membrane. While they may serve as anchoring moieties on these surfaces, these interactions differ from the activation of the integrin signaling mediated by ECM‐derived factors such as RGD.

As we previously observed high clonal ECFC expansion on RGD‐only surfaces [22], we assessed ECFC proliferation via DNA incorporation of the thymidine analog EdU (Figure 4D,E). RGD‐presenting surfaces lead to a significant increase in the proportion of EdU‐positive nuclei on surfaces, with 50‐50 RRGW‐RGD surfaces nearly doubling the fraction of proliferative cells as compared to antibody‐only surfaces. When comparing 50‐50 and 100‐0 RRGW‐RGD ratios, the presence of RRGW and immobilized antibodies on the surface did not significantly reduce ECFC proliferation, further indicating that the RGD‐cell interactions were preserved on bifunctional surfaces. Without RGD, there was no observable difference in cell proliferation compared to untreated polystyrene.

### 3.3 ECFC Capture Under Dynamic Conditions

**FIGURE 5:**
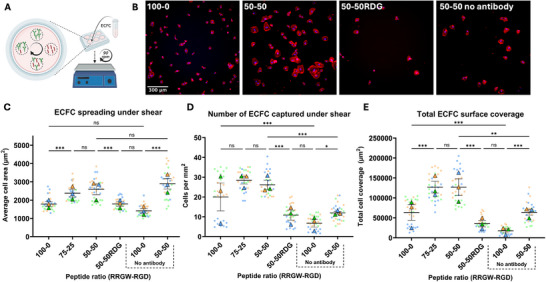
ECFC capture on bifunctional surfaces. (A) Schematic representation of the cell capture assay using an orbital shaker to induce hydrodynamic shear. (B) Fluorescence images showing captured ECFC cytoskeleton (actin in red, nuclei in blue). Number of captured cells (C), average individual cell area (D) and total surface coverage (E) obtained after 2h of dynamic capture with different surface modifications.

To determine whether the bifunctional surfaces also retained antibody‐mediated ECFC capture potential, we applied a simple orbital shaking model (Figure 5). Surfaces with anti‐CD309 immobilized via RRGW showed significantly higher numbers of ECFCs captured after 2 h under flow than surfaces with RGD only (100‐0 with anti‐CD309 applied but no Fc‐binding peptides), or controls without antibodies (50‐50 no antibody). The number of ECFCs captured was not significantly different between 100‐0 and 50‐50 RRGW‐RGD, indicating that lower surface amounts of RRGW were sufficient for anti‐CD309 immobilization and subsequent ECFC capture effects. The number of ECFCs captured was significantly higher on 50‐50 RRGW‐RGD surfaces than on 50‐50 RRGW‐RDG controls, suggesting that the presence of RGD in addition to antibodies improves ECFC retention efficiency after initial antibody interactions.

As ECFCs were captured by RRGW‐immobilized antibodies, RGD peptides present on bifunctional surfaces maintained their effect on ECFC spreading. Overall surface coverage by cells was significantly higher on bifunctional surfaces as compared to antibody‐only surfaces. This suggests the presence of an additive effect from both the immobilized antibodies and the biomimetic RGD sequence. Surfaces functionalized with a mixture of RRGW and RGD peptides without antibodies resulted in an increased total cell area despite fewer cells adhered. This is supported by the enhanced spreading induced by the presence of RGD alone (Figure 5C). These findings corroborate that RGD‐mediated cell adhesion effects were maintained on bifunctional surfaces. A concentration‐dependent effect of the RRGW peptide on ECFC capture efficiency was also observed, with a concentration of at least 10 µM required for efficient capture (Figure S5). In addition, a plateau in ECFC capture efficiency was observed at 100 µM RRGW under dynamic conditions (Figure S5). Overall, the mechanisms underlying the observed capture could be explained by a combination of rapid initial antibody‐mediated cell capture followed by integrin‐mediated firm arrest and adhesion induced by the RGD peptide.

**FIGURE 6:**
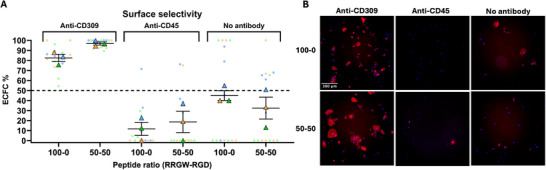
Competitive cell capture from a mixture of ECFCs and PBMCs on bifunctional surfaces. (A) Surface selectivity was determined by measuring the ratio of CD31^+^ cells. The dotted line at 50% indicates the composition of the initial ECFC/PBMC suspension. (B) Fluorescence images showing captured cells on surfaces with or without types of anti‐CD309 (to target ECFCs) or anti‐CD45 (to target PBMCs). CD31 immunostaining (red) was used to identify captured ECFCs and nucleic acid staining (blue) served as a counterstain.

As the RGD sequence is known to interact with various cell types among PBMCs [23], we next assessed whether bifunctional surfaces would maintain ECFC capture selectivity (Figure 6). We repeated the ECFC capture experiment using different antibodies targeting either the CD45 antigen, largely expressed by PBMCs, or CD309 expressed by ECFCs. As expected, ECFC populations did not interact with the anti‐CD45 antibody as we showed that they do not express this marker based on flow cytometry (Figure S1). We seeded a 50‐50 mixture of ECFCs and PBMCs together on our modified surfaces under dynamic conditions and stained adhered cells using an anti‐CD31 antibody to identify ECFCs. In the absence of antibodies, very few cells were captured on surfaces and the fraction of CD31^+^ cells was not significantly different from the initial composition of 50% – as expected for non‐specific binding. Antibody‐only as well as bifunctional surfaces successfully captured target cell types, showing significant enrichment. High cell selectivity was observed for both 100‐0 and 50‐50 RRGW‐RGD surfaces with anti‐CD309, indicating that the presence of the RGD motif doesn't significantly affect the capture selectivity provided by antibodies. The change in cell type captured based on the antibody applied suggests that bifunctional surfaces could be easily designed to target various cell types, without changing the overall peptide grafting strategy – simply by applying different antibodies.

### 3.4 ECFC Capture on Bifunctional Microcarriers

**FIGURE 7:**
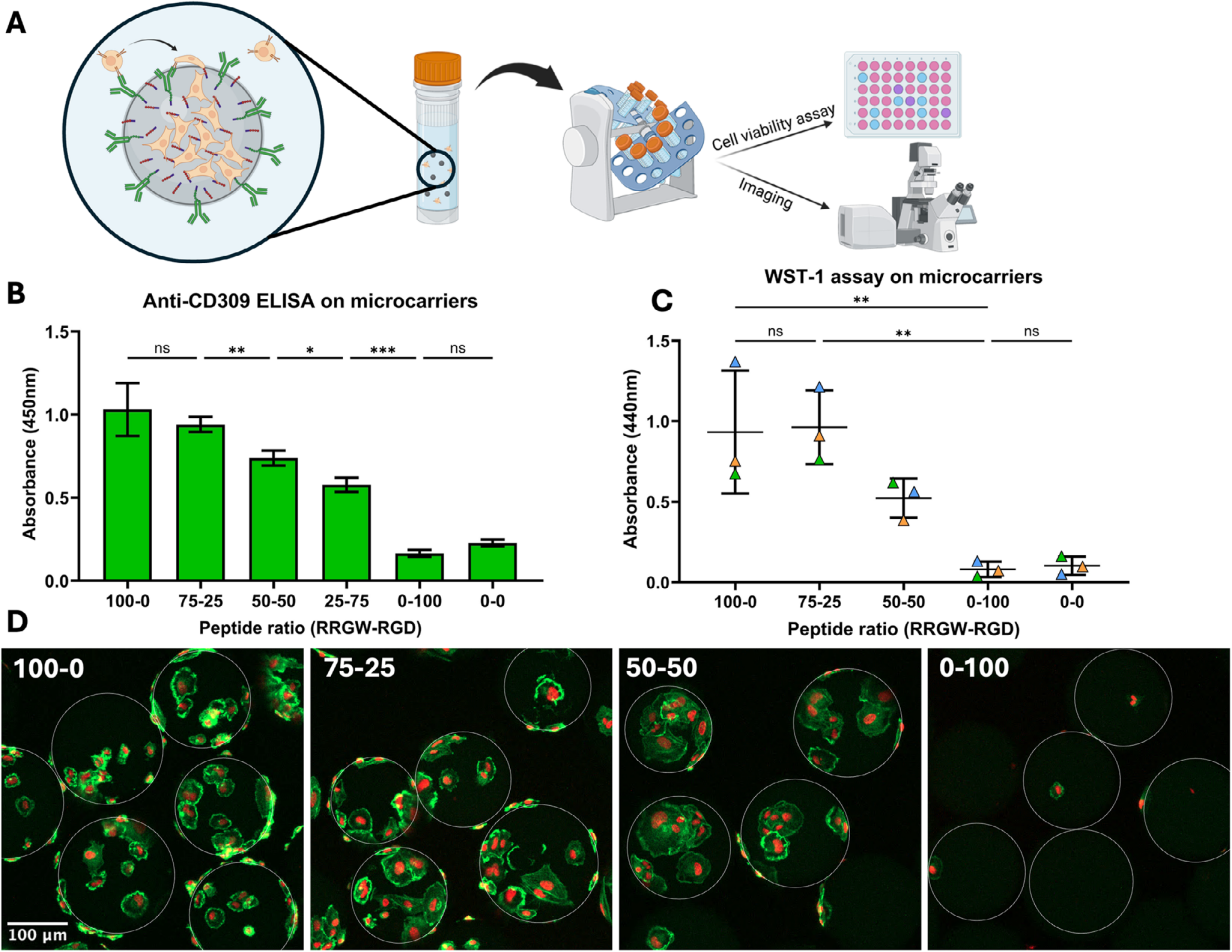
Design of a microcarrier prototype for ECFC capture using bifunctional polystyrene beads. (A) Schematic representation of the dynamic cell capture protocol on bifunctional microbeads, followed by viability testing and confocal imaging. (B) Anti‐CD309 ELISA performed on microbeads treated with different peptide mixtures showing a proportional relationship between the amount of immobilized antibodies and the peptide ratio used for surface modification (n=4 replicates). (C) WST‐1 cell viability assay performed 24h after cell capture. (n=3 donors). (D) Confocal fluorescence images of functionalized microbeads after ECFC capture, with actin (green) and nuclei (blue) staining to observe cytoskeletal organization of ECFCs adhered on the beads. Microbeads are circled in white for clarity.

Finally, a case study was conducted to evaluate the applicability of our bifunctional surface modification to 3D cell culture devices. As microcarriers are widely used to expand adherent cells in large‐scale bioreactors, aminated polystyrene beads were used as scaffold to design a bifunctional microcarrier prototype (Figure 7). Successful immobilization of antibodies and peptides on polystyrene microbeads was assessed via an adapted anti‐CD309 detection ELISA (Figure 7B). As expected, higher anti‐CD309 ELISA signals were observed on surfaces with higher RRGW–RGD ratios (Figure 7B). Next, the short‐term capture efficiency of ECFCs on bifunctional beads was evaluated through fluorescence imaging and metabolic assays to estimate adhered cell densities. Based on metabolic activity, no significant cell capture was observed on the untreated and RGD‐only microcarriers (Figure 7C). As the anti‐CD309 surface concentration increased with the RRGW ratios, the metabolic activity also increased, suggesting that higher numbers of cells were captured. This observation was further supported by the confocal images of the beads (Figure 7D), with very few adhered cells observed on beads with RGD alone in contrast to the bifunctional microcarriers showing many captured cells. While challenging to quantify in a 3D context, qualitative observations suggest that bifunctional beads induced higher ECFC spreading compared to beads with anti‐CD309 alone. While not statistically significant, a reduction in ECFC capture efficiency was observed between 75‐25 and 50‐50 RRGW‐RGD conditions. One possible explanation could be that higher RGD surface concentrations lead to more rapid ECFC spreading, reducing the surface area available for ECFC capture during the 1h incubation period. When comparing results obtained with ECFCs in dynamic culture conditions, 75‐25 RRGW‐RGD surfaces show consistent promise in mediating selective ECFC capture, adhesion and proliferation as compared to antibody‐only or RGD‐only conditions.

## 4 Discussion

In this paper, we present a novel surface modification strategy to create bifunctional surfaces with cell‐interacting antibodies and peptides. Surface concentrations of cell‐specific antibodies and cell surface receptor‐binding peptides can be controlled through simple changes in the ratio of Fc‐binding to biomimetic peptides in solution. Different antibodies (anti‐CD309 vs anti‐CD45) or cell receptor‐binding peptides (RGD vs RDG) can readily be applied to change the types of cells captured under flow, and their subsequent surface interactions. Notably, synergies between functionalities can enhance cell‐surface interactions by targeting different adhesion pathways. This strategy could be of significant interest in many regenerative medicine and biomaterials application.

A key element of the bifunctional surface modification method is the use of Fc‐binding peptides of similar molecular weight as cell‐interacting peptides, both containing a single cysteine residue to enable efficient and oriented surface grafting via click chemistry. This strategy provides a high degree of control over the surface composition, as highlighted by ELISA and fluorescence measurements. Primary amine availability on the Corning aminated polystyrene substrate was previously quantified using chemical derivatization and XPS analysis, corresponding to approximately 1.4 at.% nitrogen attributable to surface‐accessible primary amines [18]. Although atomic percentages obtained by XPS cannot be directly converted into an exact molecular surface density without assumptions regarding sampling depth, this measurement confirms the presence of reactive sites available for Sulfo‐SMCC coupling. Importantly, peptide grafting efficiency and antibody immobilization were reproducible across all substrates used in this study, despite noticeable differences in surface‐accessible primary amine density (Figure S6), indicating that amine availability was not a limiting factor for bifunctional modification under the reaction conditions applied. Nonetheless, future studies could further investigate nanoscale ligand distribution and spacing using techniques such as atomic force microscopy or surface plasmon resonance to better resolve spatial organization.

We previously immobilized various antibodies on surfaces via a recombinant cysteine‐protein G [17]. However, we were unable to apply this strategy to generate controllable and reproducible antibody‐RGD bifunctional surfaces because of the high adsorption of protein G on polystyrene compared to the smaller RGD peptide. Similar challenges would likely be encountered with direct antibody surface grafting because of a lack of control over antibody orientation and the 150‐fold molecular weight difference between immunoglobulins and short peptide sequences on the surface. Importantly, the Fc‐binding RRGW motif allows for oriented antibody immobilisation [24] which has been shown to be a critical parameter for efficient antigen recognition [25]. This approach is also easily scalable, as it does not involve extensive chemical modifications described in other antibody immobilization protocols, such as polysaccharide oxidation on the Fc region [26] or site‐specific biotinylation [27, 28]. Despite potential concerns regarding the reversible nature of peptide‐antibody interactions, our ELISA assay demonstrated sustained antigen binding on polystyrene surfaces for at least seven days in static cell culture conditions (Figure S4), consistent with the reported high affinity of the RRGW motif for mouse IgG [24]. To further enhance the stability of antibody immobilization under shear (e.g. in dynamic bioreactor systems), the RRGW peptide sequence could be modified to incorporate an additional covalent stabilization strategy enabling post‐binding crosslinking, following a “bait‐and‐hook” model [29].

The initial platform selected to evaluate the potential of this technology was ECFC selection and culture on widely used polystyrene substrates. ECFC‐based therapies have demonstrated substantial potential for treating chronic ischemic conditions by promoting tissue revascularization and functional recovery [30, 31, 32]. With growing interest in ECFC‐based therapies, the standardized 2D culture methods used may soon become insufficient to provide ECFCs in quantities and quality required for clinical applications. To our knowledge, no studies has so far reported an *ex vivo* expansion of ECFCs in a bioreactor system.

Advances in ECFC characterization have underscored the importance of identifying specific surface markers and functional assays to isolate ECFCs from other peripheral blood mononuclear cells (PBMCs) [9, 33]. Surface marker phenotyping and functional assays are now readily performed to characterize the different EC lineages, leading to a consensus on the specific surface markers expressed by ECFCs [9]. Nonetheless, the scarcity and variability of peripheral blood ECFCs remain substantial barriers to therapeutic application. ECM proteins such as collagen or fibronectin have traditionally been used for ECFC isolation from peripheral blood [13, 34], but these substrates do not provide active and selective cell capture, leading to lengthy expansion protocols. In contrast, antibody‐functionalized surfaces have been shown to rapidly and selectively capture ECFCs but are typically insufficient for promoting long‐term cell growth and proliferation and require a separate cell expansion process [14]. Our strategy addresses these limitations by simultaneously enabling selective ECFC capture via oriented anti‐CD309 antibodies immobilized through the Fc‐binding RRGW peptide, while promoting enhanced cell spreading and proliferation through an integrin‐binding RGD motif.

One potential limitation of our approach was that antibodies immobilized via RRGW motifs may mask underlying RGD peptides, thereby hindering integrin‐RGD interactions. However, both biological functionalities modified surface were effectively observed on our surfaces under both static and dynamic conditions. In accordance with the results of our previous study, the immobilization of anti‐CD309 via the RRGW sequence enabled selective ECFC capture from a mixture of PBMCs [18], as opposed to anti‐CD45 antibodies which enriched the surface in PBMCs. Additionally, ECFC spreading and proliferation induced by RGD signaling remained unaffected by the additional antibody immobilization. In dynamic culture conditions on flat substrates, a significant additive effect between anti‐CD309 mediated ECFC capture and RGD‐mediated cell spreading was observed based on the extent of surface coverage (Figure 5). The optimal RRGW peptide concentration for ECFC capture under dynamic conditions was evaluated at 100 µM (Figure S5). Consequently, RRGW–RGD ratios containing less than 50 µM RRGW were not included in capture experiments, as reduced RRGW content would be expected to compromise efficient initial cell capture. The underlying biomolecular mechanisms supporting these observations are likely based on the combination of high‐affinity VEGFR‐2 receptor‐antibody binding, promoting initial cell capture, followed by integrin‐mediated interactions through RGD motifs. Previous work showed that such interactions trigger almost instantaneous integrin clustering, leading to the nucleation of focal adhesion point formation and significant cytoskeleton reorganization with actin remodeling [35, 36, 37]. This is supported by our fluorescence imaging results, demonstrating clear differences in cytoskeletal organization and cell morphology between bifunctional and control surfaces without RGD (Figure 4).

As a proof‐of‐concept for industrial‐scale translation, we successfully applied our bifunctional surface technology to 3D microcarrier systems, widely used for large‐scale cell expansion (Figure 7). The initial prototype was successful in capturing ECFCs under rotational mixing and maintaining cellular metabolic activity. With microcarriers, no significant differences in cell surface coverage were observed, which could be due to challenges in precise cell surface area quantification on microbeads, or due to differences in the underlying polystyrene material. Further investigation of the impact of hydrodynamics and surface modifications on cell capture and long‐term expansion are required to optimize bifunctional microcarriers as a scale‐up platform. Bifunctional microcarriers could reduce ECFC handling steps by combining cell selection and expansion. Streamlined and upscaled ECFC culture on microcarriers could improve both cell yield and quality, as it has been reported with other anchorage‐dependent cell types such as mesenchymal stem cells [38].

Theoretically, the bifunctional surface modification scheme is compatible with a wide variety of applications by targeting different cell types and bioactive functionalities. We previously demonstrated that different IgGs can be immobilized on surfaces via the RRGW motif [18], and other Fc‐binding peptides could be used to target other antibody isotypes such as human IgGs [39]. Instead of Fc‐binding peptides, other small bifunctional molecules (with one terminus for covalent surface grafting) could be implemented to immobilize antibodies, such as aptamers [40]. Instead of RGD, other ECM‐derived peptides or other cell receptor binding peptides could be applied, such as the REDV sequence [41]. Depending on the application, additional functionalities can be added to the surface by combining peptides with anti‐microbial [42] or anti‐thrombotic [43] properties, for instance. Our surface modification strategy could also be transferred to different aminated materials, or materials with functional groups conducive to covalent surface grafting chemistry. For example, we previously applied ECM‐derived peptides to glass [37], polylactic acid [44] or polytetrafluoroethylene [45] surfaces to promote fast endothelialization. Bifunctional surface modifications could potentially be applied to blood‐contacting devices, including coronary stents and synthetic vascular grafts. For transplantation applications, the functionality and immunogenicity of grafted biomolecules in more complex media such as blood fractions should be further investigated.

## 5 Conclusions

In conclusion, we developed a robust, scalable and tunable bifunctional surface modification strategy with a wide range of applications in biomedical material engineering. Using click chemistry to simultaneous graft Fc‐binding peptides with biomimetic peptides, highly tunable surfaces presenting oriented antibodies with cell‐interacting peptides can be engineered. This platform enabled selective anti‐CD309 antibody mediated ECFC capture from flow, combined with RGD‐mediated cell spreading and proliferation, on the same culture substrate. Preliminary results from microcarrier‐based 3D systems further support scalability and clinical translation potential. This versatile platform offers substantial promise for applications in regenerative medicine and biomaterials design.

## Author Contributions

H.A.L. and M.‐A.C. performed conceptualization, methodology, validation, formal analysis, investigation, wrote the original draft, wrote, review and edited the final manuscript. M.A.E. performed conceptualization, methodology, wrote, review and edited the final manuscript. J.‐F.T. performed conceptualization, funding acquisition, wrote, review and edited the final manuscript. G.L. performed conceptualization, wrote, review and edited the final manuscript. C.A.H. performed conceptualization, resources, wrote the original draft, supervision, project administration, funding acquisition, wrote, review and edited the final manuscript.

## Conflicts of Interest

Mohamed A. Elkhodiry, Corinne A. Hoesli, Gaétan Laroche are listed as co‐inventors on patent WO/2022/011466, co‐owned by McGill University and Laval University, that covers the bifunctional surface technology described in this article. Hugo A. Level, Marc‐Antoine Campeau and Corinne A. Hoesli are co‐founders and equity holders of Capcyte Biotherapeutics Inc., a company holding an exclusive worldwide license to commercialize the technology.

## Supporting information




**Supporting File**: adhm71103‐sup‐0001‐SuppMat.docx.

## Data Availability

The data that support the findings of this study are available from the corresponding author upon reasonable request.
