# Supplementary material for: Bifunctional Surfaces With Immobilized Antibodies and Bioactive Peptides Mediate Selective Capture and Proliferation of Endothelial Colony‐Forming Cells
Source: Adv Healthc Mater. 2026 Apr 9;15(21):e05092. doi: 10.1002/adhm.202505092 (PMC13241468; doi:10.1002/adhm.202505092)
Supplement: Supplementary file 1 — Supporting File: adhm71103‐sup‐0001‐SuppMat.docx. [file ADHM-15-0-s001.docx]

Supplementary Information

Bifunctional surfaces with immobilized antibodies and bioactive peptides mediate selective capture and proliferation of endothelial colony-forming cells

Hugo A. Level^a#^, Marc-Antoine Campeau^a#^, Mohamed A. Elkhodiry^a^, Gaétan Laroche^b^, Jean-François Tanguay, Corinne A. Hoesli* ^a,d^

^a^ Department of Chemical Engineering, McGill University, Canada

^b^ Centre de Recherche du CHU de Québec & Département de Génie des Mines, des

Matériaux et de la Métallurgie, Université Laval, Canada

^c^ Montreal Heart Institute, Canada

^d^ Department of Biomedical Engineering, McGill University, Canada

^#^ Equal contribution

*Corresponding author

Supplementary Table

Table S1. Reagents used for flow cytometry experiments for ECFC surface marker assessment.

Supplementary Figures


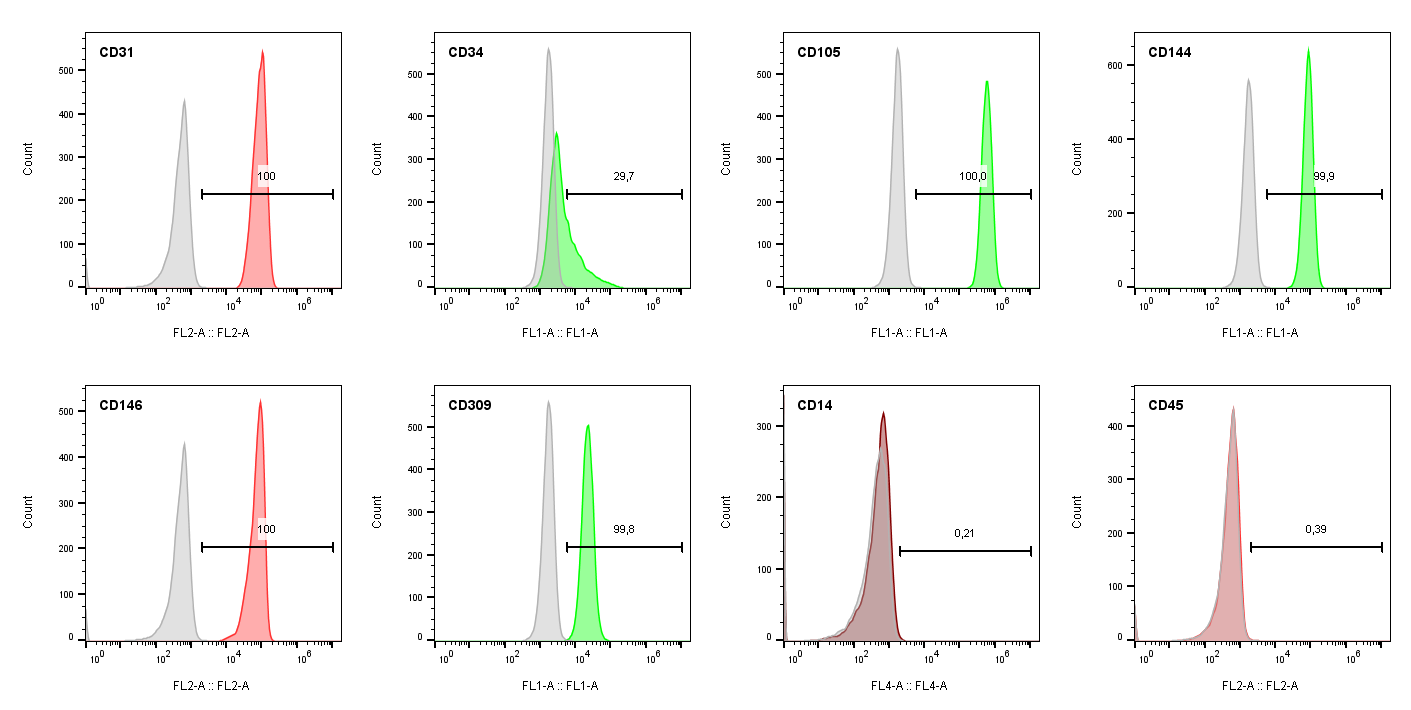


**Figure S1.** **Surface marker assessment of ECFC isolated from adult peripheral blood.** Representative flow cytometry histograms illustrating the expression of various ECFC-associated surface markers (CD31, CD34, CD105, CD144, CD146, CD309). CD14 (monocyte marker) and CD45 (hematopoietic marker) expression were used as negative controls. All reagents and stains used for this experiment are summarized in Table S1.


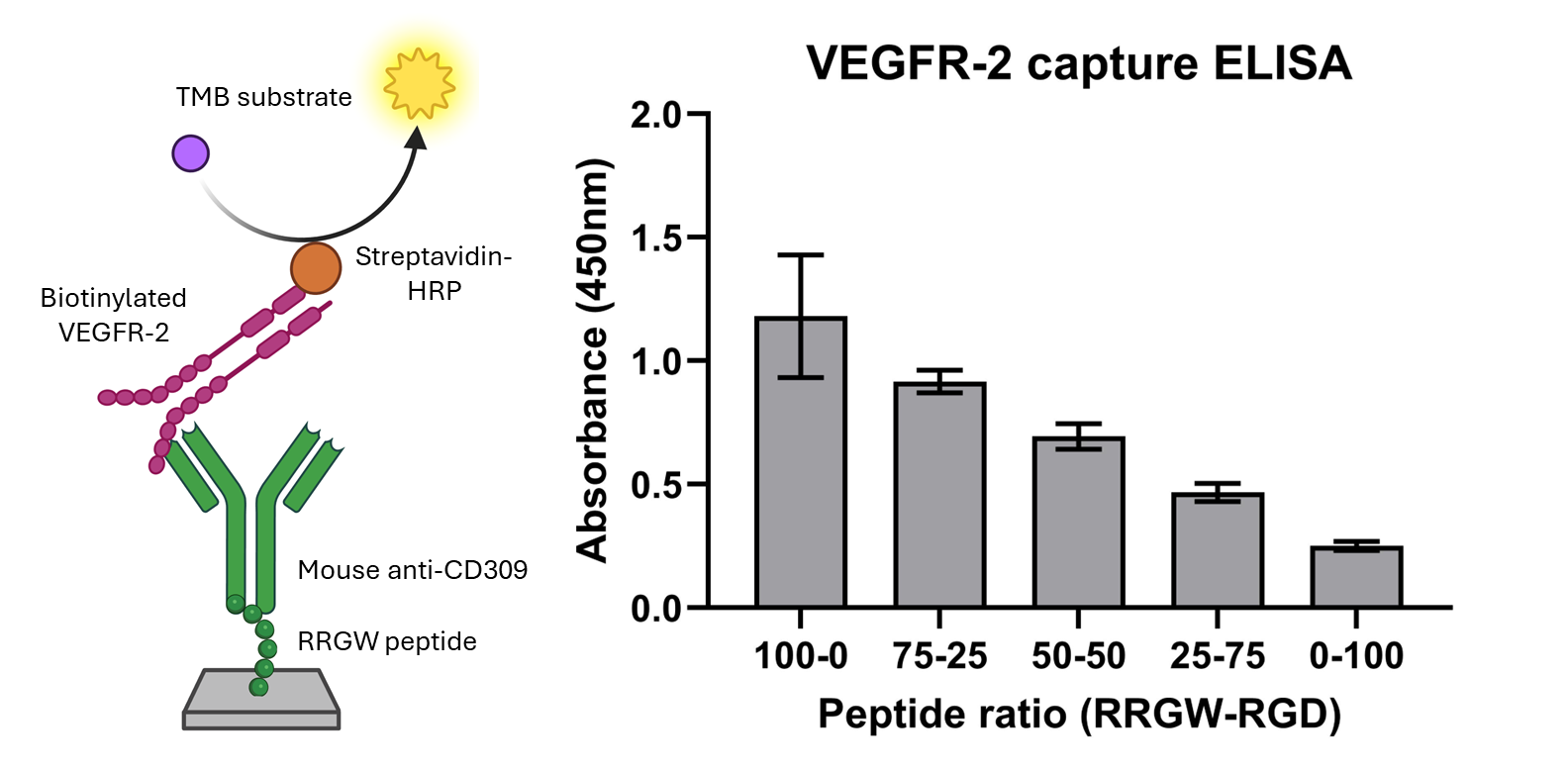


**Figure S2. VEFGR-2 capture ELISA on bifunctional surfaces**. Antigen recognition efficiency was assessed on bifunctional surfaces prepared at different peptide ratios with a modified ELISA protocol. A 0.5 ug/mL recombinant biotinylated VEGFR-2 solution was applied on the modified surfaces after immobilization of anti-CD309 antibodies. Streptavidin-HRP was then used to detect the captured VEGFR-2 antigen. The measured absorbance signal varied linearly (p<0.001) with the amount of RRGW peptide used in solution during surface grafting.


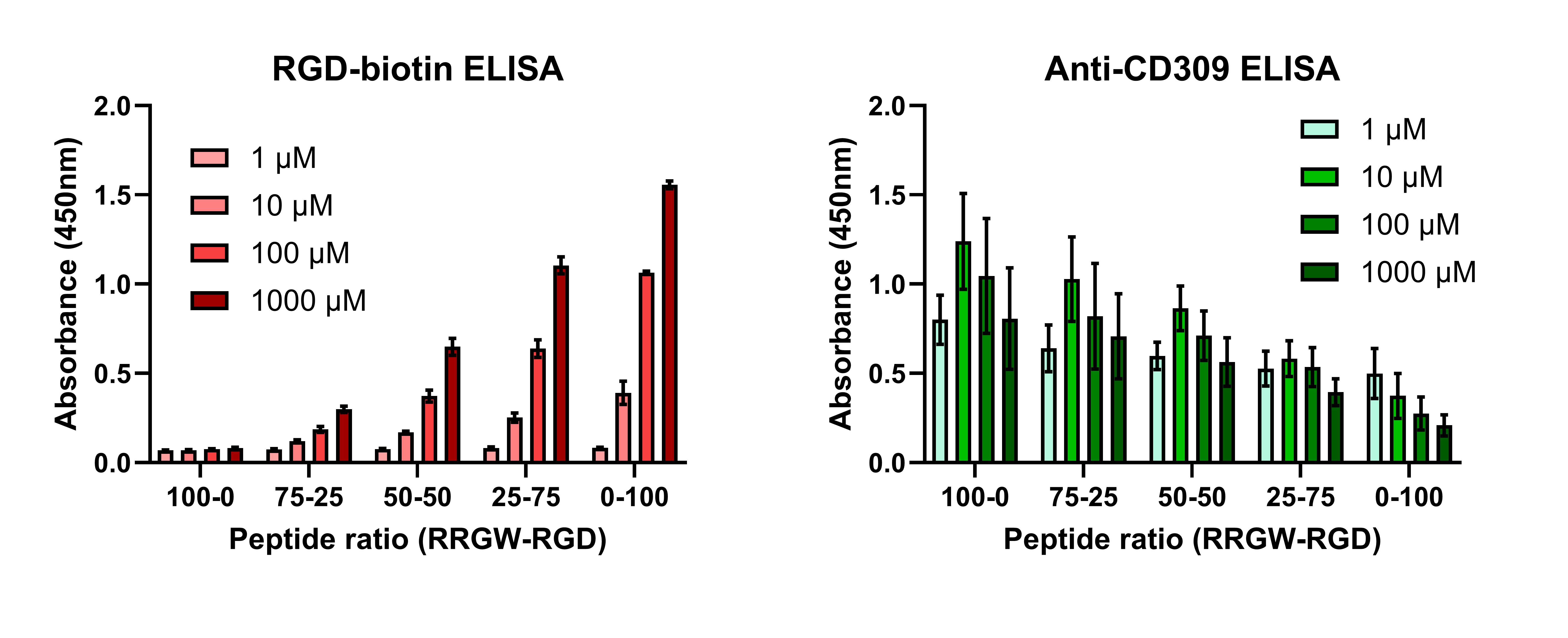


**Figure S3. Surface characterization of bifunctional surfaces for various peptide ratios and concentrations.** Polystyrene surfaces were modified using varying peptide ratios and concentrations (1–1000 μM). ELISA was used to assess the amount of surface-grafted RGD-biotin (left) and immobilized anti-CD309 (right) following functionalization.


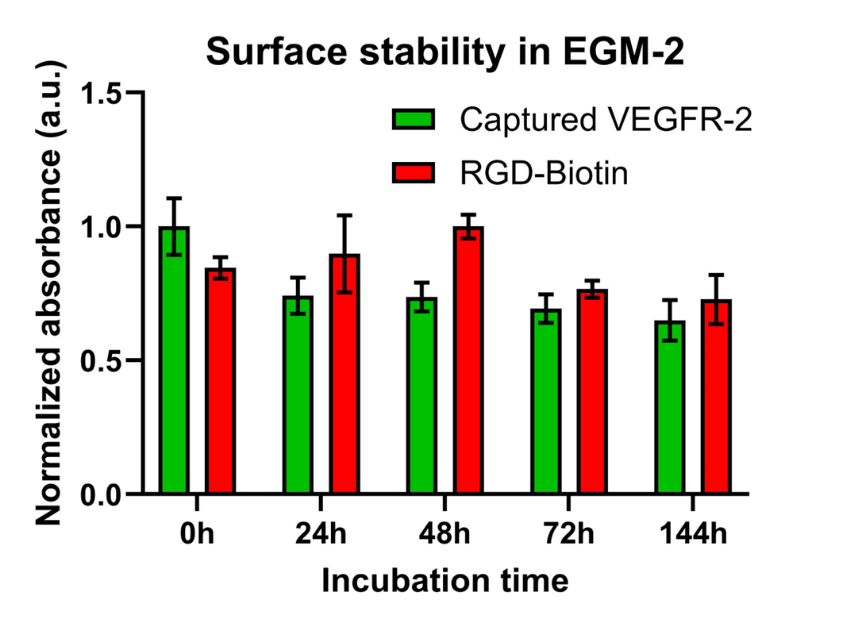


**Figure S4. Stability of bifunctional surfaces in EGM-2 culture medium, assessed by RGD-biotin and VEGFR-2 capture ELISA.** Polystyrene surfaces were modified using a 50-50 RRGW-RGD peptide ratio (100 μM total concentration) and pre-treated with 70% ethanol to minimize contamination risks.


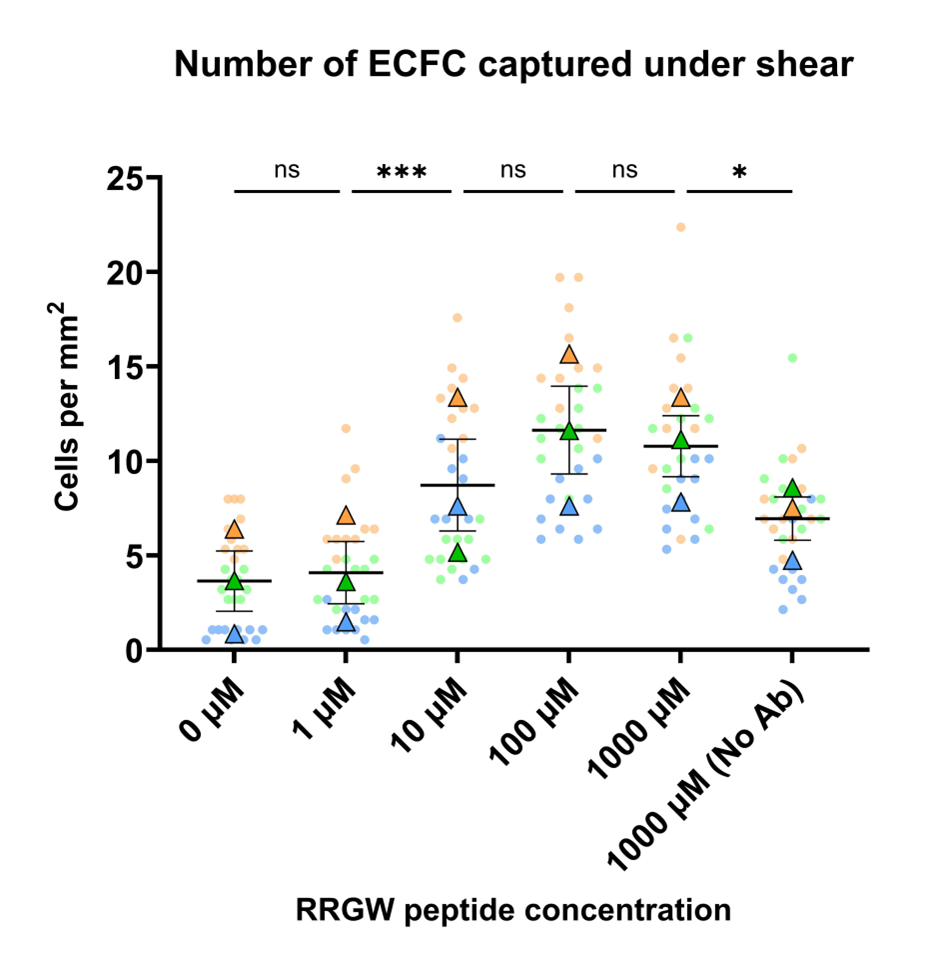


**Figure S5. ECFC capture on monofunctional surfaces treated with different concentrations of RRGW peptide.** ECFC capture was performed under shear with 3 different donors (n=3). A condition without anti-CD309 antibodies was used as a control. *** p<0.001, * p<0.05


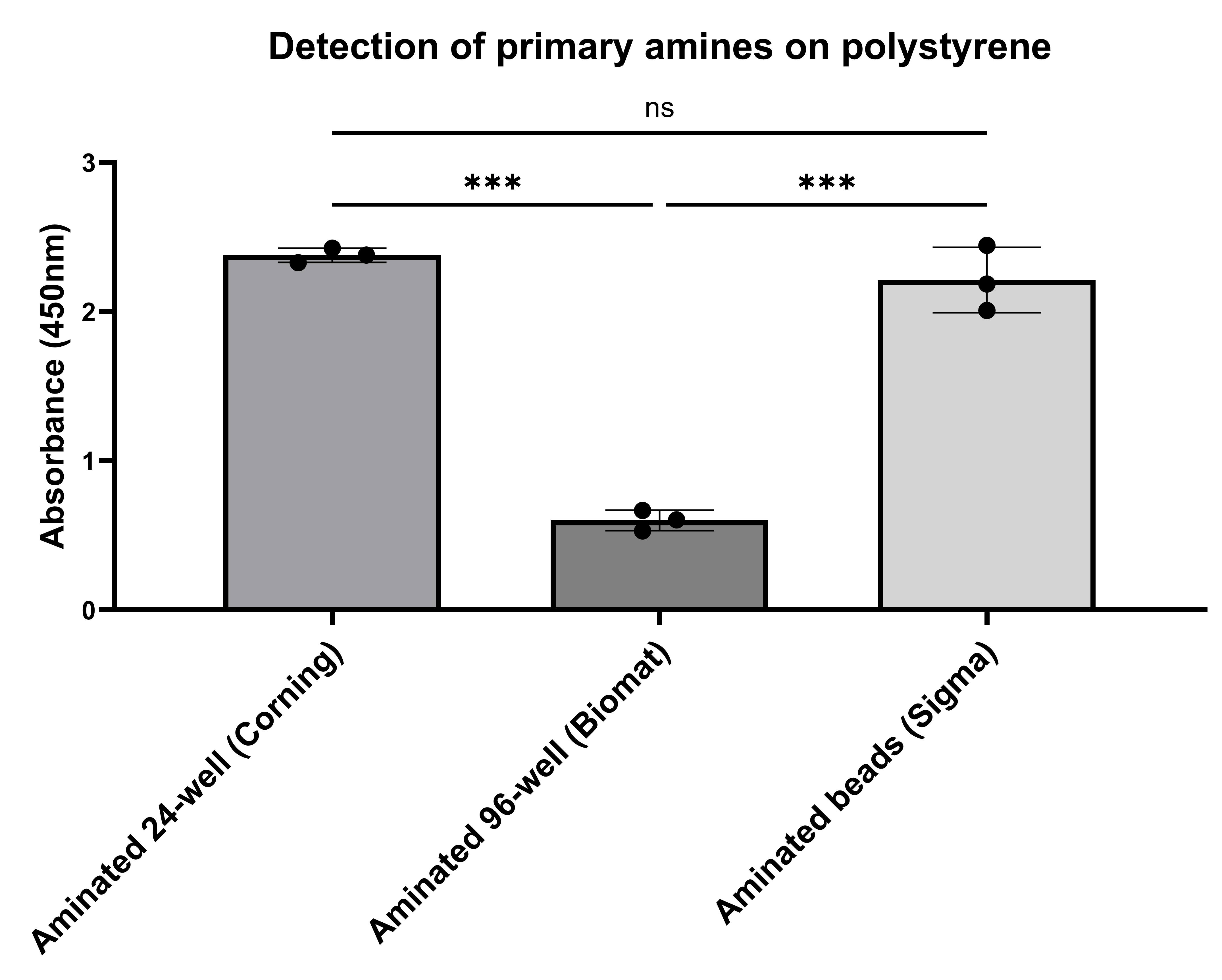


**Figure S6. Detection of primary amines on polystyrene surfaces from different sources (Corning, Biomat, Sigma).** Surfaces were reacted with 1 μg/mL of Sulfo NHS-LC-LC-Biotin (Thermo, cat. 21338) in DPBS for 2h followed by 30 min incubation with streptavidin-HRP (0.04ug/mL, Abcam; ab7403). Color development was performed using TMB, with the reagent volume normalized to surface area (100µL/cm²) to ensure comparable conditions across all substrates. *** p<0.001
